# When Proteomics Reveals Unsuspected Roles: The Plastoglobule Example

**DOI:** 10.3389/fpls.2013.00114

**Published:** 2013-04-25

**Authors:** Houda Nacir, Claire Bréhélin

**Affiliations:** ^1^Laboratoire de Biogenèse Membranaire, CNRSVillenave d’Ornon, France; ^2^Laboratoire de Biogenèse Membranaire, Université de BordeauxVillenave d’Ornon, France

**Keywords:** plastoglobule, proteomics, *Arabidopsis*, plastids, stress, subcellular fractionation, fibrillin

## Abstract

Plastoglobules are globular compartments found in plastids. Before initial proteomic studies were published, these particles were often viewed as passive lipid droplets whose unique role was to store lipids coming from the thylakoid turn-over, or to accumulate carotenoids in the chromoplasts. Yet, two proteomic studies, published concomitantly, suggested for the first time that plastoglobules are more than “junk cupboards” for lipids. Indeed, both studies demonstrated that plastoglobules do not only include structural proteins belonging to the plastoglobulin/fibrillin family, but also contain active enzymes. The specific plastoglobule localization of these enzymes has been confirmed by different approaches such as immunogold localization and GFP protein fusions, thus providing evidence that plastoglobules actively participate in diverse pathways of plastid metabolism. These proteomic studies have been the basis for numerous recent works investigating plastoglobule function. However, a lot still needs to be discovered about the molecular composition and the role of plastoglobules. In this chapter, we will describe how the proteomic approaches have launched new perspectives on plastoglobule functions.

## Introduction

**Figure 1:**
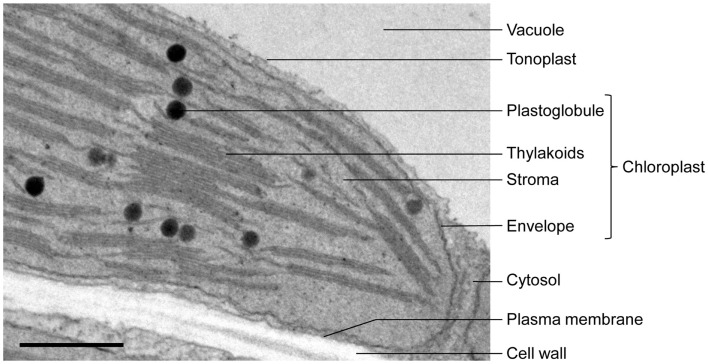
**Electron micrograph of an *Arabidopsis* leaf chloroplast showing plastoglobules in proximity with thylakoids**. Bar: 500 nm.

In addition to the network of thylakoid membranes which are the site of photosynthesis, plastids contain in their soluble phase, the stroma, some enigmatic lipoprotein bodies named the plastoglobules (cf. Figure 1). Plastoglobules can be found in diverse types of plastids, from proplastids (for review, see Nagata et al., 2002) to gerontoplasts (Kovacs et al., 2008) or etioplasts (Seyyedi et al., 1999). Although the origin of plastoglobules remains unclear, they may be closely linked to thylakoid development and dismantlement. Indeed, it has been observed that plastoglobule abundance increases when photosynthetic activity of green tissues decreases and thylakoids break down, like for example in senescent chloroplasts (Lichtenthaler, 1968; Guiamét et al., 1999; Ghosh et al., 2001), or during fruit maturation and ripening, when chloroplasts turn into chromoplasts and thylakoids disintegrate (Deruere et al., 1994; Vishnevetsky et al., 1999; Bonora et al., 2000). Reciprocally, plastoglobules are thought to be lipid reservoirs in greening tissue (Kessler et al., 1999), allowing the rapid formation of thylakoids. For example they may be involved in the formation of thylakoid membranes in de-etiolating plastids: etioplasts with poorly developed thylakoids have more plastoglobules than chloroplasts, but the plastoglobule abundance decreases during thylakoid biogenesis (Sprey and Lichtenthaler, 1966; Lichtenthaler and Peveling, 1967; Lichtenthaler, 1968). A tomographic study showing that plastoglobules are physically linked to thylakoid membranes (Austin et al., 2006) reinforced the idea that plastoglobules and thylakoids indeed share a functional relationship. The physical connection between the two compartments would allow channeling of molecules in both directions.

Plastoglobules are composed of an outer polar lipid monolayer containing neutral lipids (mainly prenylquinones, triacylglycerol, and carotenoids), and harbor proteins (for review, see Bréhélin et al., 2007; Bréhélin and Kessler, 2008). The diameter of plastoglobules is around 50–100 nm but they can enlarge to several micrometers (Thomson and Platt, 1973) depending on various factors such as plant species, plastid types, developmental stages, and environmental conditions. Numerous studies have described an increase of plastoglobule size and/or number under various environmental conditions (for review, see Bréhélin et al., 2007; Bréhélin and Kessler, 2008), such as drought (Rey et al., 2000), salt stress (Locy et al., 1996; Ben Khaled et al., 2003), or in the presence of heavy metals (Baszynski et al., 1980). Based on these ultrastructural observations, the involvement of the plastoglobules in plant responses to stress has been suggested, but biochemical or physiological evidence is missing. The exact role of plastoglobules in plant adaptation to stresses remains poorly understood. Yet, advances are being made in understanding some of their functions, mostly thanks to proteomics.

## Deciphering the Nature and Roles of Plastoglobules: From Ultrastructural Based Speculations to Proteomic Indications

The progress made in plant electron microscopy allowed the first descriptions of plastoglobules: Hodge et al. (1955) observed the presence of “dense spherical bodies” in stroma of maize mesophyll chloroplasts while Falk (1960) reported the existence in *Ficus elastica* chloroplasts of “osmiophilic spheres” and “magnoglobuli” ranging from 0.13 to 2.5 μm in diameter. Menke (1962) stated that the chemical composition of the “spherical inclusions known as osmiophilic granules or globules” was unknown, but that they were made of ether-soluble compounds, thus highlighting our ignorance of the plastoglobule composition, excepted for their lipidic nature.

The first protocols for the isolation of “osmiophilic globules” were then rapidly set up (Park and Pon, 1961; Bailey and Whyborn, 1963; Greenwood et al., 1963). They all followed a similar scheme. First, integral chloroplasts were purified from other cell components by centrifugation. Next, the chloroplasts were disrupted and plastoglobules separated from chloroplast membranes by differential centrifugation, thanks to their relatively low density. The subcellular fractionation of plastoglobules enabled scientists to investigate their chemical nature, especially with regard to their lipid and pigment contents (Bailey and Whyborn, 1963; Greenwood et al., 1963; Lichtenthaler, 1969). These studies reported the presence, in chloroplast plastoglobules, of several prenylquinones (tocopherol, phylloquinone, plastoquinone) while no significant amounts of carotenoids were detected.

While purification protocols were rapidly and easily set up, making purified plastoglobules available, the protein composition of this compartment has only started to be investigated 30 years later. Indeed, plastoglobules were long thought to be passive lipid droplets, accumulating pigments, and lipids originating from thylakoid disintegration (Smith et al., 2000). One of the first evidence for the association of proteins with plastoglobules came with the immunogold labeling of geranylgeranyl pyrophosphate synthase (GGPPS) in *Capsicum* fruits by Cheniclet et al. (1992). The authors described the presence of a pool of GGPPS around the plastoglobules. However, GGPPS is a functionally soluble enzyme and its specific physical association with plastoglobules was never confirmed. Pozueta-Romero et al. (1997) demonstrated that a major protein of bell pepper chromoplasts, the fibrillin, was a genuine component of plastoglobules and was located at their periphery. This protein was previously called fibrillin because of its high abundance in fibrils, a specialized structure of some chromoplasts wherein carotenoids accumulate (Deruere et al., 1994). It was proposed that fibrillin could built a compatible interface between the hydrophobic core of plastoglobule and the surrounding hydrophilic stroma, thereby allowing the maintenance of their structure and preventing them from coalescence (Deruere et al., 1994; Rey et al., 2000; Simkin et al., 2007). Afterward Kessler et al. (1999) showed that plastoglobules contained at least a dozen of different proteins which they named plastoglobulins. They characterized one of these plastoglobulins and showed that it belonged to the fibrillin family. Thus at the end of the twentieth century, plastoglobules were still generally viewed as passive lipid bags delimited by a coat of proteins whose nature and function were unknown. In this respect, proteomics allowed important improvement in the understanding of plastoglobule function, providing the first evidence for the presence of active enzymes in this compartment.

In 2006, two independent laboratories established for the first time the proteome of plastoglobules. While hundreds of proteins are usually listed in subcellular proteomic studies (Wienkoop et al., 2010), only as few as 30 proteins (cf. Table 1) were identified in *Arabidopsis thaliana* chloroplast plastoglobules (Vidi et al., 2006; Ytterberg et al., 2006), implying that plastoglobules are highly specialized sites dedicated to a restricted set of tasks in plastids. As expected, a major part of the proteome was constituted by proteins belonging to the plastoglobulin/PAP/fibrillin family, and another part was composed of proteins with unknown function. More astonishing, was the identification of about 10 known or putative metabolic enzymes, suggesting an active role for plastoglobules in some plastid metabolic pathways.

**Table 1:** **The chloroplast plastoglobule proteome determined by different proteomic studies**.

Accession number	Name/description	Percent mass PG core^a^	Prot. reference^b^	Confirmed plastoglobule location^c^
At4g04020	AtPGL35/FBN1a; Stress tolerance (Youssef et al., 2010)	16.1	Vidi et al. (2006), Ytterberg et al. (2006), Lundquist et al. (2012b)	GFP-fusion (Vidi et al., 2006), immunogold (Ytterberg et al., 2006)
At3g23400	AtPGL30.4/FBN4; Plastoglobule development, stress tolerance (Singh et al., 2010)	11.9	Vidi et al. (2006), Ytterberg et al. (2006), Lundquist et al. (2012b)	GFP-fusion (Vidi et al., 2006)
At4g22240	AtPGL33/FBN1b; Stress tolerance (Youssef et al., 2010)	9.6	Vidi et al. (2006), Ytterberg et al. (2006), Lundquist et al. (2012b)	
At2g35490	AtPGL40/FBN2; Stress tolerance (Youssef et al., 2010)	7.1	Vidi et al. (2006), Ytterberg et al. (2006), Lundquist et al. (2012b)	
At3g58010	AtPGL34/FBN7a	3.5	Vidi et al. (2006), Ytterberg et al. (2006), Lundquist et al. (2012b)	GFP-fusion (Vidi et al., 2006, 2007)
At2g42130	AtPGL30/FBN7b	2.1	Vidi et al. (2006), Ytterberg et al. (2006), Lundquist et al. (2012b)	
At2g46910	AtPGL31/FBN8	1.8	Ytterberg et al. (2006), Lundquist et al. (2012b)	
At5g05200	ABC1 kinase 9; Lundquist et al. (2012a)	5.2	Vidi et al. (2006), Ytterberg et al. (2006), Lundquist et al. (2012b)	
At4g31390	ABC1 kinase 1/AtACDO1; Photooxidative stress tolerance (Yang et al., 2012)	4.5	Ytterberg et al. (2006), Lundquist et al. (2012b)	
At1g79600	ABC1 kinase 3; Lundquist et al. (2012a)	4.3	Vidi et al. (2006), Ytterberg et al. (2006), Lundquist et al. (2012b)	
At3g24190	ABC1 kinase 6; Lundquist et al. (2012a)	2.6	Lundquist et al. (2012b)	
At1g71810	ABC1 kinase 5; Lundquist et al. (2012a)	1.7	Ytterberg et al. (2006), Lundquist et al. (2012b)	
At3g07700	ABC1 kinase 7; Lundquist et al. (2012a)	0.8	Lundquist et al. (2012b)	
At4g19170	Carotenoid cleavage dioxygenase (AtCDD4)	3.3	Vidi et al. (2006), Ytterberg et al. (2006), Lundquist et al. (2012b)	
	Carotenoid catabolism (Huang et al., 2009)	
At4g32770	Tocopherol cyclase (VTE1); Vitamin E biosynthesis (Porfirova et al., 2002)	2.6	Vidi et al. (2006), Ytterberg et al. (2006), Lundquist et al. (2012b)	GFP-fusion (Vidi et al., 2006), immunogold (Ytterberg et al., 2006)
At5g08740	NAD(P)H dehydrogenase C1 (NDC1); Phylloquinone synthesis, plastoquinone reduction	2.5	Vidi et al. (2006), Ytterberg et al. (2006), Lundquist et al. (2012b)	GFP-fusion (Eugeni Piller et al., 2011)
At1g54570	PES1; Phytyl ester synthesis (Lippold et al., 2012)	2.6	Vidi et al. (2006), Ytterberg et al. (2006), Lundquist et al. (2012b)	
At3g26840	PES2; Phytyl ester synthesis (Lippold et al., 2012)	1.4	Vidi et al. (2006), Ytterberg et al. (2006), Lundquist et al. (2012b)	
At1g78140	Protein with Methyltransferase type 11 domain-1	1.5	Vidi et al. (2006), Ytterberg et al. (2006), Lundquist et al. (2012b)	
At2g41040	Protein with Methyltransferase type 11 domain-2	1.5	Vidi et al. (2006), Ytterberg et al. (2006), Lundquist et al. (2012b)	
At1g32220	Protein with NAD-dependent epimerase/dehydratase domain	2.1	Vidi et al. (2006), Ytterberg et al. (2006), Lundquist et al. (2012b)	
At1g06690	Protein with aldo-keto reductase domain	1.5	Vidi et al. (2006), Ytterberg et al. (2006), Lundquist et al. (2012b)	
At4g39730	Lipase/lipooxygenase, PLAT/LH2 family protein	1.6	Lundquist et al. (2012b)	
At1g73750	Protein with α/β hydrolase domain	0.4	Lundquist et al. (2012b)	
At5g41120	Esterase/lipase/thioesterase family protein	0.3	Lundquist et al. (2012b)	
At5g42650	Allene oxide synthase (AOS) Jasmonic acid biosynthesis (Laudert et al., 1996)		Vidi et al. (2006), Ytterberg et al. (2006)	GFP-fusion (Vidi et al., 2006)
At2g21330	Fructose-bisphosphate aldolase-1		Vidi et al. (2006), Ytterberg et al. (2006)	GFP-fusion, enzymatic activity (Vidi et al., 2006)
At4g38970	Fructose-bisphosphate aldolase-2		Vidi et al. (2006), Ytterberg et al. (2006)	GFP-fusion, enzymatic activity (Vidi et al., 2006)
At2g01140	Putative fructose-bisphosphate aldolase-3		Vidi et al. (2006), Ytterberg et al. (2006)	
At3g10130	Protein with SOUL heme binding domain	1.8	Vidi et al. (2006), Ytterberg et al. (2006), Lundquist et al. (2012b)	
At2g34460	NAD(P)-binding Rossmann-fold superfamily protein	1.5	Vidi et al. (2006), Ytterberg et al. (2006), Lundquist et al. (2012b)	
At4g13200	Unknown protein-1	1.9	Vidi et al. (2006), Ytterberg et al. (2006), Lundquist et al. (2012b)	
At3g43540	Unknown protein-2 (DUF 1350)	1.3	Lundquist et al. (2012b)	
At3g27110	Protein with peptidase M48 domain	0.3	Lundquist et al. (2012b)	

*^a^Contribution of each protein to the protein mass of the total PG core proteome (in %) determined in Lundquist et al. (2012b). ^b^Plastoglobule proteome from which the protein was identified. ^c^Reference confirming the localization of the protein in plastoglobules*.

By combining shotgun proteomics with spectral-counting techniques, a quantitative proteomic approach has recently been applied to the plastoglobule proteome in order to detect low abundant proteins and quantify the relative abundance of each protein within plastoglobules (Lundquist et al., 2012b). By using defined selection filters (presence in biological and technical replicates, enrichment in the plastoglobule fraction, previously characterized subcellular localization), the core plastoglobule proteome was then restricted to 30 proteins. A striking observation was that despite the increased sensitivity of current mass spectrometers, the plastoglobule proteome size did not enlarge. Only seven new low abundant proteins were added to the plastoglobule proteome while others were considered as likely contaminants and therefore were removed from the previous proteome (cf. Table 1). This new proteome was established with *Arabidopsis* plants submitted to high light stress, which could explain at least part of the observed variations.

## The Protein Composition of *Arabidopsis* Chloroplast Plastoglobule *per se*

A detailed comparison of the three different plastoglobule proteomes has recently been described (Lundquist et al., 2012b). Thus this review will only briefly summarize the function of the main protein components of the plastoglobules.

### The plastoglobulin/PAP/fibrillin family

Plastoglobulins (also called PAP for Plastid-lipid-Associated Protein, or fibrillin) represent more than 50% of the protein mass of the plastoglobule core proteome in *Arabidopsis* chloroplasts (Lundquist et al., 2012b). Proteomic studies have demonstrated that several members of this family are associated with plastoglobules, some being exclusively localized in plastoglobules (Kessler et al., 1999; Austin et al., 2006; Vidi et al., 2006) while others may partition between plastoglobules, thylakoids, and stroma (Rey et al., 2000; Lundquist et al., 2012b). However it still remains to be determined if the plastoglobulin composition varies within the plastoglobule population of a single plastid and if the presence of one specific plastoglobulin defines a kind of specialization of the plastoglobules (Vidi et al., 2006). Indeed, the abundance of each plastoglobulin within the plastoglobule proteome is not uniform, ranging from 1.8 to 16.1% (Lundquist et al., 2012b), and some plastoglobulins accumulate in plastoglobules under high light conditions, while others accumulate after dark treatment (Ytterberg et al., 2006). This suggests that plastoglobulins have diverse functions. In agreement, plastoglobulin mutant phenotypes suggest an implication of some of these proteins in plant growth regulation and development, as well as in stress tolerance and disease resistance (for review, see Singh and McNellis, 2011). Notably, plastoglobulins have recently been demonstrated to be implicated in jasmonate biosynthesis (Youssef et al., 2010) or plastoquinone accumulation (Singh et al., 2010, 2012), which illustrate their role in stress tolerance. However, the exact mechanism of action of plastoglobulins still needs to be clarified.

### The tocopherol cyclase VTE1

The tocopherol cyclase (VTE1) story illustrates how proteomics can sometimes prompt us to reconsider accepted models. VTE1 catalyzes the second to last step of α-tocopherol (vitamin E) synthesis consisting of the cyclization of 2,3-dimethyl-5-phytyl-1,4-hydroquinol (DMPQ) to γ-tocopherol (Porfirova et al., 2002). Before the plastoglobule proteomic studies were published, it was generally thought that the entire pathway for the vitamin E biosynthesis was taking place at the plastid envelope membrane (Soll et al., 1985). However, proteomics, coupled with immunolocalization and GFP-fusion studies, have demonstrated a specific localization of VTE1 in plastoglobules (Austin et al., 2006; Vidi et al., 2006; Ytterberg et al., 2006; Lundquist et al., 2012b). In addition to DMPQ, VTE1 also catalyzes the conversion of plastoquinone (PQH_2_-9), another prenyl quinone, into plastochromanol (PC-8) (Szymanska and Kruk, 2010; Zbierzak et al., 2010). Both substrates (DMPQ and PQH_2_-9) and products (γ-tocopherol and PC-8) are present at least partially in plastoglobules (Vidi et al., 2006; Zbierzak et al., 2010), providing an additional evidence for the implication of plastoglobules in the synthesis of prenyl quinones. Tocopherols, as well as plastoquinone and plastochromanol have antioxidant activity, exerting a photoprotective role to thylakoid lipids and photosystem II (Eugeni Piller et al., 2012). Thus, plastoglobules may represent an antioxidant reservoir available to protect the thylakoid membranes from oxidative stress.

### The NAD(P)H quinone dehydrogenase C1 (NDC1)

When the first plastoglobule proteomes were published, the function of NDC1 was unknown, and its localization was believed to be mitochondrial (Michalecka et al., 2003). The identification of NDC1 in the plastoglobule proteome prompted Kessler and colleagues to investigate its localization and function (Eugeni Piller et al., 2011). Its localization in plastoglobules was confirmed by means of GFP-fusion constructs and its dual localization in plastoglobules and mitochondria demonstrated by western blot analysis. The authors also showed that in the knock-out *ndc1* *Arabidopsis* mutant, the plastoquinone pool was more oxidized than in the wild type, demonstrating that NDC1 is involved in the regeneration of reduced plastoquinone. In addition, the *ndc1* mutant was almost totally deprived of phylloquinone (vitamin K_1_), suggesting that the enzyme plays a part in the phylloquinone production.

### The carotenoid cleavage dioxygenase 4

Carotenoid cleavage dioxygenase 4 (CCD4) (also named NCED4 for 9-cis epoxy-carotenoid dioxygenase 4) has been reported to occur in the plastoglobule proteomes (Vidi et al., 2006; Ytterberg et al., 2006; Lundquist et al., 2012b). The members of the CCD family cleave different carotenoids and xanthophylls to apocarotenoids such as abscisic acid. *In vitro*, AtCCD4 cleaves preferentially the apocarotenoid 8′-apo-β-caroten-8′ to yield β-ionone (Huang et al., 2009). However, the *in planta* substrate for this enzyme has not been discovered yet, and its current function in plastoglobule is unknown. The stable isotope experiments performed by Ytterberg et al. (2006) showed an accumulation of the enzyme after dark treatment compared to high light treatment, suggesting an implication of AtCCD4 in carotenoid breakdown.

### ABC1 kinases

Six members of the activity of BC1 (ABC1 complex) kinases have been identified in plastoglobules, representing the second most abundant protein family of the plastoglobule proteome (Lundquist et al., 2012b). The ABC1 kinases belong to the atypical protein kinase superfamily. In *Arabidopsis*, this superfamily is composed of 15 members, among which six are most likely mitochondrial and the remaining ones are supposed to be targeted to plastids (Lundquist et al., 2012a). An ABC1 kinase was first described as playing an essential role in electron transfer in the bc1 complex of *Saccharomyces cerevisiae* (Bousquet et al., 1991). Two plastidial ABC1 kinases, AtOSA1, and AtACDO1 which locates to plastoglobules, were proposed to act against photooxidative stress (Jasinski et al., 2008; Yang et al., 2012). However, the function of the plant ABC1 kinases is unknown, and the significance of the localization of the six members of this family in plastoglobules, representing 18% of the protein plastoglobule mass, is still mysterious.

### PES1 and 2

Plastoglobules were demonstrated to be the site of accumulation of fatty acid phytyl esters (FAPEs) under stress conditions or during senescence (Ischebeck et al., 2006; Gaude et al., 2007). FAPEs consist of a phytol molecule, originating from the breakdown of chlorophyll, esterified to an acyl group removed from galactolipids. The accumulation of FAPEs in plastoglobules is believed to prevent the membranes from the detergent-like properties of free phytol and acyl groups (Bréhélin and Kessler, 2008). The enzyme(s) responsible for this synthesis were initially unknown. However, two putative acyltransferases with sequence similarities to esterases/lipases/thioesterases (At1g54570 and At3g26840) were identified in the plastoglobule core proteome (Vidi et al., 2006; Ytterberg et al., 2006; Lundquist et al., 2012b). Lippold et al. (2012) therefore hypothesized that these enzymes could be involved in the FAPE synthesis. Using reverse genetic and heterologous expression approaches, they demonstrated that indeed both proteins catalyzed the formation of FAPEs during stress conditions, and were therefore likely involved in the maintenance of the photosynthetic membrane integrity.

### Other proteins

Three isoforms of fructose-1,6-biphosphate aldolases (FBPA), and the allene oxide synthase (AOS) were identified in the two first published proteomes (Vidi et al., 2006; Ytterberg et al., 2006) but excluded from the plastoglobule proteome established by Lundquist et al. (2012b). AOS is implicated in jasmonate synthesis (Schaller and Stintzi, 2009), while FBPA participates to the Calvin cycle and glycolysis. The localization in plastoglobules of the four enzymes was confirmed by transient expression of GFP-tagged constructs (Vidi et al., 2006) and an FBPA activity was measured in the plastoglobule fractions (Vidi et al., 2006). These enzymes were removed from the “core plastoglobule proteome” because they were not enriched in the plastoglobule fraction. They could however partition between plastoglobules and other compartments of the chloroplast. These four enzymes should perhaps not be excluded from the plastoglobule proteome but rather be considered as enzymes with roles in plastoglobules as well as in other plastid compartments.

Some additional low abundant proteins were described in plastoglobules. The majority was represented by proteins with unknown function, such as two proteins with methyltransferase domains, a SOUL heme binding protein, or newly identified proteins (Lundquist et al., 2012b) such as the M48 metalloprotease. There is no doubt that the understanding of their function will reveal another panel of the plastoglobule story.

## Other Plastoglobule Proteomes

The specialized structures wherein carotenoids accumulate during chromoplastogenesis define the morphology of chromoplasts (reviewed in Egea et al., 2010). For instance, during globular chromoplast formation, carotenoids accumulate in plastoglobules, leading to an increase of plastoglobule size and/or number (Jeffery et al., 2012). Ytterberg et al. (2006) analyzed the proteome of red pepper chromoplast plastoglobules and showed that it contains (i) plastoglobulins, one of the most abundant proteins of pepper chromoplasts (Siddique et al., 2006), which are known to be involved in carotenoid sequestration (for review, see Bréhélin and Kessler, 2008; Egea et al., 2010), (ii) enzymes already characterized in plastoglobules such as VTE1 and FBPA, and (iii) enzymes involved in carotenoid synthesis including ζ-carotene desaturase (ZDS), lycopene β-cyclase (LYCB), and two β-carotene β-hydroxylases. In addition, phytoene synthases were proposed to locate to plastoglobules based on evidence from GFP-fusion localization experiments (Shumskaya et al., 2012). The presence of such enzymes suggests that plastoglobules are not only involved in the sequestration of carotenoids but also in carotenoid biosynthesis. However uncertainty still persists about the localization of ZDS and LYCB since they were characterized in envelope fraction of *Arabidopsis* chloroplasts by spectral-counting proteomics (Joyard et al., 2009). The possibility remains that the envelope fraction was contaminated with plastoglobules in these latter experiments. Yet, in the same study, plastoglobulins were not found in the envelope fraction but in the thylakoids, suggesting that plastoglobules are rather associated with the thylakoid membranes in this preparation. Another explanation could be that the localization of these enzymes differs depending on plastid type (chloroplast or chromoplast), organ (leaf or fruit), or species. This underlines the need for other chromoplast plastoglobule proteomic studies.

Finally, other proteomic data about plastoglobules could be taken from the proteome of *Chlamydomonas reinhardtii* eyespot. The eyespot apparatus is believed to play the role of a directional light sensor (reviewed in Kreimer, 2009). It is constituted by two layers of carotenoid-rich globules associated with thylakoids. Transmission electron microscopy observations suggested similarities between the globules of the eyespot apparatus and plastoglobules. The resemblance of both compartments is confirmed by proteomics. Indeed, the proteomes of *Chlamydomonas reinhardtii* eyespot and plastoglobules contain common homologous proteins, such as proteins with plastoglobulin domain, ABC1 kinases, or FBPA (Schmidt et al., 2006; Kreimer, 2009). Proteins with plastoglobulin domains may prevent the coalescence of the carotenoid-rich globules and maintain interactions with membranes. The actual function of the other proteins found both in eyespot and plastoglobules is not yet understood.

While the plastoglobule proteomes from different plastid types share proteins in common, they also contain specific proteins depending on the plastid type. This variation in the plastoglobule protein composition may indicate a possible specialization of the plastoglobule function depending on the tissues considered.

## Conclusion

Proteomic approaches have brought substantial insight into our understanding of plastoglobule functions. Our conception of the plastoglobules has dramatically changed from insipid passive lipid droplets inside plastids to particles with active role at the crossroad of diverse metabolic pathways, for example in vitamin biosynthesis. Notably, the common denominator of the vast majority of plastoglobule proteins is a possible involvement in the response to stress. Recently, our understanding of the plastoglobule function has reached an upper level with the combination of proteomics and co-expression analysis (Lundquist et al., 2012b). Assuming that a set of coexpressed genes is involved in the same or related metabolic pathway, a co-expression network of the core plastoglobule genes has been built, with the goal to provide a framework to better decipher plastoglobule roles. Four major co-expression modules were defined, with specific functions. Thus a model was proposed, where plastoglobules are involved in senescence, plastid biogenesis and proteolysis, redox regulation, photoacclimation, and isoprenoid biosynthesis (Lundquist et al., 2012b). Nevertheless, major efforts still need to be accomplished to comprehensively understand the role of plastoglobules in the plant biology, and especially their involvement in the plant responses to stress.

## Conflict of Interest Statement

The authors declare that the research was conducted in the absence of any commercial or financial relationships that could be construed as a potential conflict of interest.
